# Reviews of New Books

**Published:** 1890-08

**Authors:** 


### Book Notices.

THE PRACTICAL APPLICATION OF ELECTRICITY IN MEDICINE AND SURGEY. By J. A. Liebig, Jr., Ph. D., and Geo. H. Rohé, M. D. Profusely illustrated; 333 pages. F. E. Davis, publisher. Price $2, net.

A book of great value to all who would practice the electro-therapeutic art. It is eminently practical and devoid of all theory except that demanded by way of explanation. Part I. treats in a concise manner of the fundamental laws of electricity and magnetism, and decribes the various batteries and cells. Part II. of electro-physiology and diagnosis, with full descriptions of electro-medical batteries. Part III. of the general therapeutic effects of electricity and methods of application and special electro-therapeutics. Altogether the book is an intelligent account of the science of electricity and a trustworthy guide to its application.

B. F. C.

A HANDBOOK OF DERMATOLOGY.—For the Use of Students. By A. H. Ohmann-Dumesnil, A.M., M. D., Professor of Dermatology in the St. Louis College of Physicians and Surgeons, etc. Illustrated, 160 pages. St. Louis: St. Louis Medical and Surgical Publishing Co.

This small hand-book is intended as a guide to students in their reading, especially those attending lectures at the St. Louis College of Physicians and Surgeons. It is a succinct practical little hand-book. B.

